# Association between psoas abscess and prosthetic hip infection: a case-control study

**DOI:** 10.3109/17453670902947424

**Published:** 2009-04-01

**Authors:** Frédéric-Antoine Dauchy, Michel Dupon, Hervé Dutronc, Bertille de Barbeyrac, Sylvie Lawson-Ayayi, Vincent Dubuisson, Vincent Souillac

**Affiliations:** ^1^Department of Infectious Diseases Tropical Medicine, Hôpital Pellegrin, CHU de Bordeaux, Université Bordeaux 2France; ^2^Microbiology Laboratory, Hôpital Pellegrin, CHU de BordeauxFrance; ^3^Institut de Santé Publique d’Epidémiologie et de Développement (ISPED), Institut National de la Santé et de la Recherche Médicale Unité 593 (INSERM)France; ^4^Department of General Surgery, Hôpital Pellegrin, CHU de BordeauxFrance; ^5^Department of Orthopaedic Surgery, Hôpital Pellegrin, CHU de Bordeaux, Université Bordeaux 2France

## Abstract

**Background and purpose** The relationship between prosthetic hip infection and a psoas abscess is poorly documented. We determined the frequency of prosthetic hip infections associated with psoas abscesses and identified their determinants.

**Methods** We conducted a 2-year observational study. Data from patients with psoas abscesses that were associated with prosthetic hip infections were examined in a case-control study.

**Results** Of 106 patients admitted to the Infectious Diseases Department with prosthetic hip infection, 13 also had a psoas abscess (12%; 95% CI: 6–19). By conditional logistic regression analysis, psoas abscesses were observed more frequently in cases of hematogenous prosthetic infections (OR = 93, p = 0.06) and in patients with a history of neoplasm (OR = 20, p = 0.03).

**Interpretation** Our results suggest that the presence of psoas abscesses is a frequent but under-diagnosed complication of prosthetic hip infection. We recommend that an abdominal CT scan be performed on patients with hematogenous prosthetic hip infection or with a history of neoplasm.

The risk of infectious complications after hip replacement is 1–2%, even despite the use of standard operating procedures including laminar airflow and routine antimicrobial prophylaxis (Widmer 2001). Most authors recommend removal of the device to eradicate chronic infections.

A prosthetic hip infection can give rise to a psoas abscess, which can occur hematogenously or by microbial inoculation (Ricci et al. 1986). Primary psoas abscesses are caused by a hematogenous agent from another, distant infection. Secondary infections are caused by contiguous infections. The iliopsoas bursa is the largest synovial bursa in the body and is connected to the hip joint in 14% of subjects (Chandler 1950). In addition, the new capsule formed after prosthetic implantation can interact with the iliopsoas bursa (Steinbach et al. 1985). There have been few reports on psoas abscess associated with an infected prosthetic hip (for review, see Buttaro et al. 2002).

Because of the limited information on the association between psoas abscesses and prosthetic hip infection, we assessed its frequency and tried to identify its causes.

## Patients and methods

We retroactively studied patients treated for hip implant infection between 1 January 2005 and 31 December 2006 at our Infectious Diseases Department, which is a tertiary referee center. We examined postoperative and hematogenous prosthetic infections. The clinical history of the patient was used to predict orthopedic implant infections, and the diagnosis had to be confirmed using bone biopsies. When coagulase-negative staphylococcus was suspected to be present, at least 3 positive samples were required for a positive diagnosis. Three usual types of prosthetic infections were distinguished (Widmer 2001): early and late postoperative prosthetic infections were defined as prosthetic-related infections occurring less than 3 months and more than 3 months after surgery, respectively, with progressive joint-function impairment. Hematogenous prosthetic infection was defined as a delayed infection surrounding the prosthetic hip with sudden onset of pain and fever, usually occurring more than 2 years after surgery. Presence of bacteremia was noted.

We determined whether cases of prosthetic hip infection were associated with a psoas abscess. A psoas abscess was defined as a white blood cell-containing fluid, collecting within the fascia of the psoas muscle. In this study, the observation of a psoas abscess on a CT scan was required to confirm the diagnosis. It was not necessary to have conclusive bacterial identification of the psoas drainage fluid, since previous antibiotic use could have given a false-negative result. Nevertheless, bacterial identification was noted when obtained.

In general, our patients were monitored for prosthetic- related infections for at least 2 years following initial treatment. Because of the short time period of this study, complete remission was unobtainable for each patient. We considered a patient cured when there were no clinical signs of inflammation, there was no radiological progression, and there was normalization of inflammatory parameters. The date of the patient’s last visit was noted.

### Data analysis and statistics

Data analysis was conducted in 2 stages. In the first stage, descriptive analysis of the population was done. In the second stage, data from patients with psoas abscesses that were associated with prosthetic hip infection were compared to data from sex-matched patients with prosthetic hip infections treated during the same time period. For each confirmed case, we randomly selected 3 control subjects. Cases and control subjects were matched on a 1-to-3 basis. Statistical analysis was done using STATA software version 6.0 (StataCorp, College station, TX). A conditional logistic regression analysis was performed to identify clinical or biological factors independently associated with psoas abscesses. Variables with a p-value below 0.25 in univariate analysis were entered into the logistic regression model. Association of psoas abscesses with a given variable was estimated by matched odds ratio (OR). The level of significance was set at p #x003C; 0.05. The study was not powerful enough to allow investigation of the statistical association between abscess and rare factors such as liver disease, rheumatoid arthritis, or peripheral vascular disease.

**Figure:**
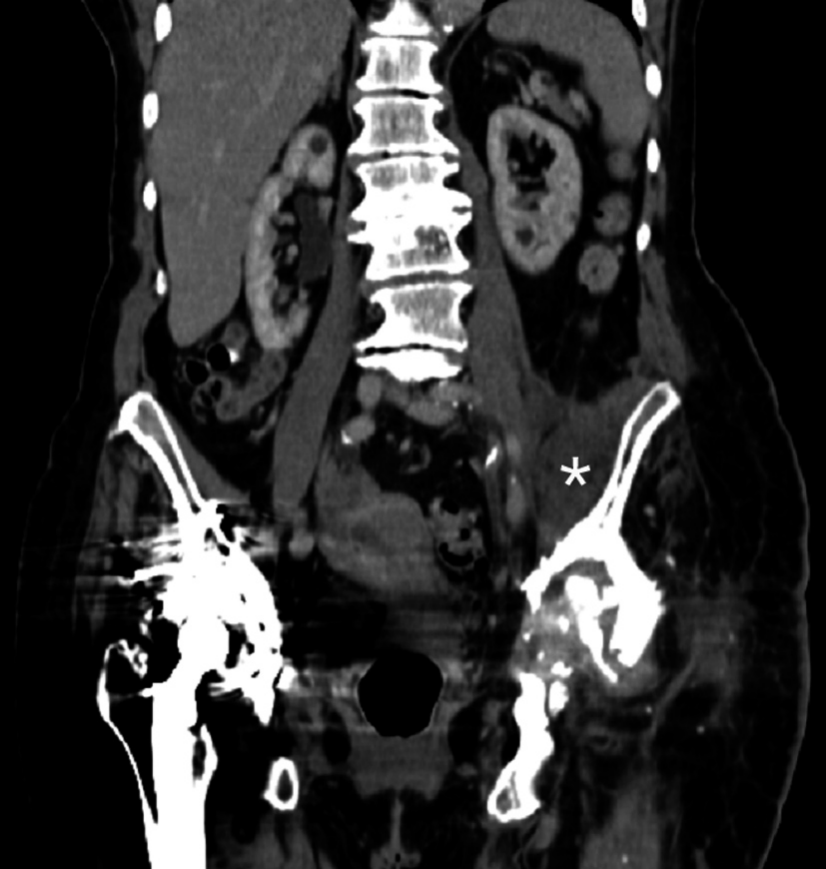
CT scan with soft tissue window showing a 7 × 5 cm left psoas abscess (asterisk). The left infected implant has been removed.

## Results

We found 13 patients with psoas abscesses that were associated with prosthetic hip infection (Table 1). These abscesses were adjacent to the hip joint and involved either the iliac muscle alone, or both the iliac and the psoas muscles (Figure). The mean size of the abscess was 7 (3–14) cm. No vertebral osteomyelitis was observed. 6 patients had a clinical history of neoplasm (lung: 2; throat: 1; liver: 1; kidney: 1; bladder: 1). Surgical or radiological drainage was performed on 10 patients; the 3 other patients declined the drainage procedure. Of 7 patients who had hematogenous prosthetic infection, 3 had positive hemocultures.

**Table 1.** Case-control study: univariate analysis

Characteristics	Patients (n = 13)	Controls (n = 39)	p-value
Males/females	9/4	27/12	
Mean age (SD)	70 (10)	67 (11)	0.4
Neoplasm, no.	6	4	0.02
Immunosuppressant therapy, no.	1	5	0.6
Crohn’s disease, no.	0	0	
Time in weeks between implantation			
and diagnosis of prosthetic			
infection, mean (SD)	114 (98)	18 (28)	0.01
Type of prosthetic infection, no.			0.009
early postoperative infection	1	9	
late postoperative infection	5	25	
hematogenous infection	7	5	
Clinical findings			
fever, no.	8	15	0.2
right/left side, no.	7/6	20/19	0.9
Size in cm of psoas abscess, mean	7	–	
(range)	(3–14)	–	
Culture of peroperative samples, no.			0.8
*Staphylococcus aureus*	5	10	
coagulase-negative staphylococcus	3	16	
streptococcus	3	7	
Gram-negative bacteria	2	6	
Positive culture from psoas, no.			
(among 10 patients)	8	–	
Positive blood culture, no.	3	2	0.09
Treatment			
drainage procedure of psoas			
abscess, no.			
radiological	6	–	
surgical	4	–	
none	3	–	
surgical revision of prosthetic hip, no.			0.7
debridement and prosthetic retention	4	9	
one-stage reimplantation	0	4	
two-stage reimplantation	7	17	
removal without reimplantation	2	9	
duration of antibiotics in weeks,			
mean (SD)	10 (2)	10 (2)	0.9
Outcome			
persistence of psoas abscess on			
CT, no (among 11 patients)	3	–	
remission, no.	10	29	0.9

99mTc-HMPAO-labeled white blood cell scintigraphy was performed on 7 of 13 patients. This procedure failed to diagnose the presence of an intra-abdominal abscess in all cases, presumably because the digestive organs can reduce the sensitivity of the procedure, especially on the right side (due to the caecum).

During the same period, 171 patients were admitted to our hospital with prosthetic hip infection. 150 patients (87%) had a postoperative infection and 21 patients had a hematogenous prosthetic infection. Abdominal CT imaging was performed on 106 of the 171 patients (62%) for several reasons, including abdominal pain or abnormal liver tests during hospitalization. Of the 106 patients undergoing a CT scan, 90 (85%) had a postoperative infection and 16 (15%) had a hematogenous prosthetic infection. Prosthetic hip infections were associated with psoas abscesses in 8% of the 171 patients (95% CI: 4– 12%) and in 12% of the 106 patients (95% CI: 6–19%).

Psoas abscesses were associated with hematogenous prosthetic infection (OR = 93, p = 0.06) and clinical history of neoplasm (OR = 20, p = 0.03) (Tables 1 and 2). Because of the retrospective nature of our study, conclusions cannot be drawn regarding the influence of psoas abscess identification on patient outcome. However, it is worth noting that 3 patients with psoas abscesses did not recover after removal of the prosthetic implant and that 2 had persistent psoas abscesses.

**Table 2.** Case-control study: logistic regression analysis

Full model	aOR **^a^**	95% CI	p-value
Type of prosthetic infection			
early postoperative infection (ref.)	–	–	–
late postoperative infection	4	0.27–62	0.3
hematogenous infection	93	0.85–105	0.06
Neoplasm	20	1.4–276	0.02
Fever	1.3	0.23–7.8	0.8
Positive blood culture	0.86	0.03–20	0.9

**^a^** aOR: adjusted matched odds ratio.

## Discussion

Psoas abscesses consist of a purulent collection in the iliopsoas compartment. Classical symptoms include fever and lumbar pain (Ricci et al. 1986). An association between psoas abscesses and prosthetic hip infections has been reported only rarely in the literature (Buttaro et al. 2002). Our results—that 12% of prosthetic hip infections are associated with psoas abscess—suggest that this association is under-diagnosed. One reason may be that standard radiology procedures do not identify the presence of psoas abscesses. Furthermore, CT scans are not routinely performed because of the artifacts seen around the implant area.

We believe that the presence of such abscesses has an influence on treatment. Drainage procedures were performed in 10 cases, with the aim of making sure of adequate debridement of the abscesses. Moreover, removal of the implant was performed in 5 of 7 cases of acute hematogenous infection. Furthermore, we cannot exclude the possibility that bacteria persisting in the psoas abscess might cause relapse, with reinfection of a new prosthetic implant.

We found that psoas abscesses were associated with hematogenous prosthetic hip infection. The time between implantation and diagnosis of prosthetic infection was long in the cases of psoas abscesses (p = 0.01). This finding could be due to the association between hematogenous prosthetic infection and discovery of an infection more than 2 years after surgery (Dupon et al. 2001).

The spread of infection between the implant and the iliopsoas compartment can be explained by the presence of acetabular fissures, caused by acetabular screws that have penetrated the iliac bone or by previous surgeries (Figure). The hip could also be infected by direct spread through the iliopsoas bursa. Because the iliopsoas bursa and the synovial membrane of the hip joint are in close contact, a distally dissecting psoas abscess could be a source of bacterial seed to the hip (Steinbach et al. 1985).

Finally, we believe that an abdominal CT scan should be performed on patients presenting with hematogenous prosthetic infections, defined as a delayed infection with sudden onset of pain and fever. Our results also indicate that a CT scan should be considered for patients with prosthetic infection and a clinical history of neoplasm.
